# Proficiency in identifying, managing and communicating medical errors: feasibility and validity study assessing two core competencies

**DOI:** 10.1186/s12909-016-0755-5

**Published:** 2016-09-02

**Authors:** Abd Moain Abu Dabrh, Mohammad Hassan Murad, Richard D. Newcomb, William G. Buchta, Mark W. Steffen, Zhen Wang, Amanda K. Lovett, Lawrence W. Steinkraus

**Affiliations:** 1 Division of Preventive, Occupational, and Aerospace Medicine, Mayo Clinic, 200 First Street SW, Rochester, MN 55905 USA; 2 The Evidence-based Practice Center, Mayo Clinic Robert D. and Patricia E. Kern Center for the Science of Health Care Delivery, Rochester, MN USA

**Keywords:** Communication skills, Professionalism, Core competencies, Medical training, Medical errors, ACGME

## Abstract

**Background:**

Communication skills and professionalism are two competencies in graduate medical education that are challenging to evaluate. We aimed to develop, test and validate a *de novo* instrument to evaluate these two competencies.

**Methods:**

Using an Objective Standardized Clinical Examination (OSCE) based on a medication error scenario, we developed an assessment instrument that focuses on distinctive domains [context of discussion, communication and detection of error, management of error, empathy, use of electronic medical record (EMR) and electronic medical information resources (EMIR), and global rating]. The aim was to test feasibility, acceptability, and reliability of the method.

**Results:**

Faculty and standardized patients (SPs) evaluated 56 trainees using the instrument. The inter-rater reliability of agreement between faculty was substantial (Fleiss k = 0.71) and intraclass correlation efficient was excellent (ICC = 0.80). The measured agreement between faculty and SPs evaluation of resident was lower (Fleiss k = 0.36). The instrument showed good conformity (ICC = 0.74). The majority of the trainees (75 %) had satisfactory or higher performance in all six assessed domains and 86 % found the OSCE to be realistic. Sixty percent reported not receiving feedback on EMR use and asked for subsequent training.

**Conclusion:**

An OSCE-based instrument using a medical error scenario can be used to assess competency in professionalism, communication, using EMRs and managing medical errors.

**Electronic supplementary material:**

The online version of this article (doi:10.1186/s12909-016-0755-5) contains supplementary material, which is available to authorized users.

## Background

In 2001, the Accreditation Council for Graduate Medical Education (ACGME) defined six critical competencies in graduate medical education and mandated that resident-trainees be evaluated in those competencies [1]. Educators are particularly challenged in evaluating inter-personal and communication skills and professionalism, as assessing these two competencies might be influenced by various factors, including perception and social factors [2]. Both cores have been mostly measured through using multiple evaluators (e.g., faculty, peers) and through role-playing scenarios to provide subjective nonstandardized feedback on performance [3]. Optimally, educators are able to demonstrate that graduating residents are competent in appropriate use of medical records, effective communication with patients, and effective communication with other healthcare professionals. Resident-trainees also need to demonstrate integrity, ethical behavior, accept responsibility, follow through on tasks, and demonstrate care and concern for patients [4].

Several of the elements described in the definitions of these two competencies can be assessed in a clinical encounter in which residents interact with the electronic medical record (EMR) and electronic medical information resources (EMIR), identify a medical error, communicate to the patient that an error has occurred, and provide a plan for corrective action. This scenario is not rare. Medical errors are very common and have been recognized as a major cause of morbidity, mortality and increased health expenditure [5]. Similarly, the use of EMRs is becoming a standard of care in the US. Patient safety concerns, cost-effectiveness research, and medico-legal pressures have all contributed to the drive for healthcare providers to acquire and effectively use EMRs [6–8]. The ability to rapidly and accurately access patient medical data and communicate medical data to patients can be lifesaving. Competency in using EMRs has been recently even more emphasized through the Meaningful Use Incentive Program by the Centers for Medicare and Medicaid Services [9]. The special skills required to identify and communicate an error are closely tied to both competencies; inter-personal and communication skills, as well as professionalism. These two competencies can be evaluated using the traditional methods of direct faculty observation and global assessment. However, newer methods that include simulation, the use of standardized patients (SPs) and Objective Structured Clinical Examinations (OSCEs) are particularly important in this context. Studies have reported using OSCE to evaluate professionalism [10], communication and both competencies combined [11, 12]. OSCEs have been used to teach and evaluate communication of adverse outcomes and medical errors [11, 13]. Calls for establishing and documenting competency in using EMRs have also been made by the American Medical Informatics Association and others who highlighted the impact of such competency on patient safety and quality of care [14, 15]. Although several studies found OSCE valid and reliable in various settings [16–19]; others found it not fully reliable with variable results across the six competencies [20].

The ACGME’s Residency Review Committee agreed that the initial measurement tools they developed to assess the ACGME Competencies should be revised [21]. Although many tools are available, the validity evidence, direction and educational outcomes of the majority of them are lacking [22]. In response, we planned to develop and use a *de novo* assessment instrument combined with an OSCE-like scenario as a tool in which SPs and faculty evaluate medical residents’ ability to use the EMR and available EMIRs to detect a medical error, communicate this error to patients and provide corrective action. We hypothesized that the use of this medical error-centered scenario would be a feasible and acceptable tool for testing the two competencies of professionalism and, interpersonal and communication skills. We also tested for the agreement and reliability of this instrument using different faculty and standardized patients during simulation training.

## Methods

### Setting and participants

The study was conducted at the Mayo Clinic-Rochester, a tertiary care academic center in Minnesota. The study designed as a single-station OSCE-based simulation assessment scenario. Fifty-six participants were mainly first- (PGY1), second- (PGY2) and third-year (PGY3) Internal Medicine residents doing a rotation in Preventive Medicine and Public Health, and second-year fellows in Preventive Medicine. Additionally, the study included 10 medical students (4^th^ Year MS), and eight preventive medicine fellows (PGY4 and 5). All trainees (except med students) had received orientation and training on using the EMR and had completed at least an internship year using this EMR. This simulation activity is considered by trainees as an expected regular departmental training event within didactic educational activities and clinical training or rotations; hence, trainees were not informed about the purpose of this scenario in order to achieve blinding and we did not pursue any specific sampling methodology. Each scenario was observed by preventive medicine faculty via closed circuit TV in a dedicated simulation center. Videos were captured using a secure server for concurrent or subsequent faculty viewing for duplicate evaluation. Screen shots demonstrating websites (EMIRs) visited by resident during the encounter were saved. The Information Technology Specialists and the simulation center staff created a safe electronic environment that allowed residents to access the EMR of the SP that was identical to a chart seen in usual practice. Additionally, we visually measured the time trainees spent using EMR and EMIR before meeting the SPs and during encounter, and recorded the resources (e.g. websites) they used during this training.

The study initially started with proof of concept, instrument refinement, and feasibility stages, testing the instrument on 15 residents who were assessed by 4 faculty members (AMAD, LS, MHM, RN) and one SP.

### Standardized patients (SPs) and observing faculty

Trained standardized patients participated in this scenario. These SPs have extensive experience in role-playing multiple scenarios at the simulation center. The study investigators developed the scenario and revised and reviewed it for accuracy, realism, acceptability by faculty as well as the feedback from SPs and the pilot group of 15 residents. Faculty received training on medical simulation with SPs, debriefing techniques and the use of performance checklist and global assessment instruments. Eventually, six faculty members evaluated all 56 trainees. One faculty observed the scenario in real time and provided an evaluation at the conclusion of OSCE. One of the other five faculty members subsequently reviewed the video of the same encounter, blinded to the previous rating, and provided a rating of each resident using the same instrument.

Preparatory to the OSCE, trainees received didactic and small group training on blood and body fluid exposure procedures, communication in healthcare, recommendations on use of EMR/EMIR resources, and medical error as part of their Preventive Medicine and Internal Medicine curricula. An electronic curriculum was also available as a resource and a required/recommended reading list was provided to each trainee at the beginning of their Preventive Medicine rotation.

### Encounter scenario

The scenario entailed the SP presenting as a registered nurse who has worked full time at the Emergency Department (ED) for 20 years. The nurse has been generally quite healthy and visits the clinic that day for counseling after a needle-stick incident that took place the previous day while caring for a patient with a low viral load of Human immunodeficiency virus (HIV). She had screening labs drawn and was started on post-exposure prophylaxis for 4 weeks per CDC guidelines. The nurse reports extreme fatigue and difficulty staying alert. She brought her medications in because she is concerned about side effects that might warrant a change in the regimen. She presents the trainee with two bottles of Lamivudine, Zidovudine (Combivir®) and Levetiracetam (Keppra®). The trainee is expected to consider a medication error because Keppra is not used for HIV prophylaxis and to realize that another drug with a similar name was intended to be prescribed, (Kaletra® (Lopinavir and Ritonavir)). The Keppra-Kaletra dispensing error is a recognized error according to the FDA [23, 24]. The trainee has access to a simulated EMR that is not in a production environment and to standard web resources/EMIRs to help in identifying the medication and describe drug interactions and side effects. The trainees are instructed to perform an initial history, exam and discussion. They are told that, as in the clinic, they can leave the room to staff the patient with faculty, discuss the case with the faculty, and then return to the room to dismiss the patient and provide instructions about management and follow up. Finally, they debrief with faculty.

### Assessment instrument

This ***In****strument for****Co****mmunication skills and****Pr****ofessionalism****A****ssessment* (**InCoPrA**) was developed, reviewed, pilot-tested, and revised by the study investigators, taking into consideration the ACGME definition of competencies and existing tools used for other OSCE scenarios and competencies evaluation [21, 25, 26] and the feedback provided during the pilot testing. Professionalism was defined as the commitment to carrying out professional responsibilities and an adherence to ethical principles with integrity, compassion, honesty and respect while responding to patients’ needs and managing their healthcare appropriately. Interpersonal and communication skills were defined as the ability to effectively communicate clearly, directly, thoroughly and responsibly with the patients, their families, and healthcare team members while maintain comprehensive knowledge of medical records and pertinent information.

The instrument piloting process had three forms or parts; 1) evaluation of trainees by Faculty; 2) evaluation of trainees by SP; and 3) trainee self-administered survey. The faculty and SP instrument forms use a 3-point Likert-like scale for the different questions (outstanding; satisfactory; and unsatisfactory). Six domains are addressed in the SP and faculty assessments (the context of discussion, communication and detection of error, management of the error, empathy, use of EMR and EMIR, and a global rating). Faculty and SPs are provided with a manual for the instrument that suggests questions/checklist of items that can be used to rate each category. The instrument is included with the oneline-only material (Additional file 1).

The self-evaluation survey by residents consists of asking them about how they self-rate their general skills of EMR and EMIR use, and then rating their performance after the encounter for comparison. Also, they are asked to rate how realistic the medical error scenario encounter felt, how comfortable they were using EMR and EMIR during the encounter, and how often they receive feedback from someone during their current training on the use of such resources. Additionally, they were asked to disclose if they received training using EMR or EMIR during medical school and/or residency, if they think it is helpful to receive more training, and if yes, which level they think it might be helpful.

### Statistical analyses

The pilot phase of this study was to demonstrate the acceptability and feasibility of this assessment instrument (15 residents). Later on, and after adding 41 trainees, we evaluated the inter-observer agreement to determine reproducibility, conformity and consistency between the raters (faculty and SPs) overall and over each domain. We conducted descriptive analyses and presented rates, means, and range for each item/domain where pertinent. To evaluate the inter-rater agreement, we used Fleiss’ kappa [27], in which 0.21–0.40 suggests fair agreement, 0.41–0.60 moderate agreement, 0.61–0.80 substantial agreement, and >0.81 almost perfect agreement. We also calculated the intra-class correlation coefficient (ICC) using 2-way random-effects models. ICC <0.40 can be interpreted as poor, 0.40–0.75 as fair to good, and >0.75 as excellent. All analyses were conducted using Stata version 14.0 (StataCorp LP, College Station, TX).

We described the use and statistics of EMR/EMIR usage, and trainee self-evaluation survey results were collected and presented using descriptive statistics.

### Human subject protection

The Mayo Clinic Institutional Review Board (IRB) approved the study as an educational intervention required by the respective training programs and considered it to be IRB-exempt study (45 CFR 46.101, item 1). This simulation activity is considered as an expected regular departmental training event within the didactic clinical educational activities and training, therefore no specific participation consent deemed required. The Mayo Clinic Simulation Center obtained standard consents for observation for all trainees per institutional guidelines. Additionally, the study authors and coordinators obtained standard consent for observation for all trainees per institutional guidelines.

## Results

In the pilot phase, faculty and SPs evaluated 15 trainees using the instrument. Table 1 summarizes the pilot results. In addition to the training we provided to our SP’s and faculty initially, the pilot stage served to provide feedback to our experience before enrolling more trainees.

**Table 1:** Pilot results summary of SPs and faculty evaluation on the six domains of the instrument

Category	SP assessment	Category	Faculty assessment
Context for discussion	73 % outstanding	Context for discussion	85 % outstanding
	18 % satisfactory		15 % satisfactory
	9 % unsatisfactory		0 % unsatisfactory
Communication and Management	64 % outstanding	Communication of detection of error	61 % outstanding
	36 % satisfactory		31 % satisfactory
	0 % unsatisfactory		8 % unsatisfactory
Empathy	73 % outstanding	Management of error	62 % Outstanding
	18 % satisfactory		23 % satisfactory
	9 % unsatisfactory		15 % unsatisfactory
Honesty and Truthfulness	82 % outstanding	Empathy	77 % Outstanding
	18 % satisfactory		23 % satisfactory
	0 % unsatisfactory		0 % unsatisfactory
Closure	85 % outstanding	Use of EMR and EMIR	62 % Outstanding
	15 % satisfactory		38 % satisfactory
	0 % unsatisfactory		0 % unsatisfactory
Global rating	56 % outstanding	Global rating	77 % Outstanding
	36 % satisfactory		16 % satisfactory
	18 % unsatisfactory		7 % unsatisfactory

After enrolling all 56 trainees, the majority of the trainees (75 %) had satisfactory or higher performance in all six assessed domains as shown in Table 2. The range of time spent in encounters ranged between14–47 min. In testing the reliability of the instrument, the inter-rater agreement between faculty members was substantial (Fleiss k = 0.71) and intraclass correlation efficient showed strong conformity among faculty (ICC = 0.8). The measured agreement between faculty and SPs were fair (Fleiss k = 0.39). The instrument showed moderate robustness (Pearson *r* = 0.43) and good conformity (ICC = 0.74).

**Table 2:** Final summary of results of SPs and faculty evaluation on the six domains of the instrument

Category	SP assessment^a^	Category	Faculty assessment
Context for discussion	46 % outstanding	Context for discussion	40 % outstanding
	33 % satisfactory		57 % satisfactory
	20 % unsatisfactory		3 % unsatisfactory
Honesty and Truthfulness	63 % outstanding	Communication of detection of error	41 % outstanding
	22 % satisfactory		32 % satisfactory
	15 % unsatisfactory		27 % unsatisfactory
Empathy	56 % outstanding	Empathy	77 % Outstanding
	39 % satisfactory		32 % satisfactory
	5 % unsatisfactory		1 % unsatisfactory
Closure	43 % outstanding	Management of error	62 % Outstanding
	44 % satisfactory		
			23 % satisfactory
	13 % unsatisfactory		
			15 % unsatisfactory
Global rating	48 % outstanding	Use of EMR and EMIR^b^	43 % Outstanding
	39 % satisfactory		39 % satisfactory
	13 % unsatisfactory		13 % unsatisfactory
		Global rating^c^	29 % Outstanding
			46 % satisfactory
			25 % unsatisfactory

^a^ Data were not available for 3 trainees

^b^Data were not available for 3 trainees

^c^Data were not available for 1 trainee

### EMR and EMIR usage

We collected the usage data of 46 trainees. Twenty-seven trainees opted to use EMIR to further find helpful resources while 19 trainees did not access any online resources. Eighteen of the 27 trainees used more than one resource to seek further helpful information. For those who identified the medical error (35 trainees), the average time spent using EMR before entering the room was 3 min and 23 s (based on 28/35 trainees) while they spent 80 s during the encounter (based on 35/35 trainees) using EMR.

For those who missed the medical error (11 trainees), the average time spent using EMR before entering the room was 2 min (based on 7/11 trainees) while 2 min and 41 s were spent during the encounter with a total average of 127 s (based on 11/11 trainees) using EMR. Summary of results is shown in Table 3.

**Table 3:** Summary of EMR/EMIR usage^a^

	Detected medical error group (35 trainees) TimeΩ (N of trainees based on)	Did not detect medical error (11 trainees) TimeΩ (N of trainees based on)
Average time spent using EMR before meeting SP	03:23 (28)	02:00 (7)
Average time spent using EMR during encounter	01:20 (35)	2:41 (11)
Average time spent using EMR (combined)	01:58 (28)	02:07 (7)
Average time spent using EMIR	02:22 (35)	00:52 (10)
EMIR use during encounter	None (18) Yes (17) o Micromedex® (14) o UpToDate® (9) o Google® (8) o Aids.gov® (4) o CDC^b^ (3) o WHO®^c^ (2) o Wikipedia® (2) o Other sources (4)	None (9) Yes (2) o Micromedex® (1) o Google® (1) o MedicineNet® (1)

^a^Data of 46 trainees ΩMinutes: Seconds format

^b^Centers for Disease Control and Prevention ^c^World Health Organization

### Trainees/residents self rating

After finishing each encounter, 47 trainees (84 %) returned the survey about their experience and their use of EMR and EMIR. Thirty-three trainees (70 %) found the scenario to be realistic and significantly resembling real-life scenario. While 34 of the trainees (72 %) reported receiving training on the use of EMR during residency and/or medical school training and considered themselves to have above average experience with EMR and EMIR, only 13 of them (38 %) rated their own performance that day to be matching to their self-rated experience. Twenty-eight trainees (60 %) reported the lack of feedback from staff members, senior residents or fellows about their experience with access to EMR. Nineteen trainees (40 %) stated that it will be helpful to receive short post-internship re-training courses or sessions.

## Discussion

In this study, we developed, piloted and tested the reliability of an OSCE-based instrument to evaluate professionalism and communication using a medical error scenario. The majority of residents were found by faculty and SPs to have satisfactory or higher performance in six relevant domains (the context of discussion, communication and detection of error, management of the error, empathy, use of EMR and EMIR and a global rating). The instrument showed substantial agreement and conformity between faculty raters but a fair to moderate reliability between faculty and SPs.

The majority of residents found the OSCE to be realistic. Although most of the residents received prior training in using EMR, the majority reported lack of feedback and asked for subsequent training.

Professionalism and communication are highly important for healthcare professionals to perform, evolve and establish provider-patient, as well provider-provider, relationships. The proficiency in these two competencies, however, is challenging to assess. Mazor et al. [10] highlighted the complexity of evaluating professionalism using qualitative and quantitative analyses and concluded the need for models that assess and address the inter-rater reliability and diversity in viewing professionalism. Medical simulation has become an accepted means of safely training healthcare providers and preparing them for real life scenarios; examples include physical examination and interviewing skills, procedural testing, and communication of test results or clinical recommendations. With *The Next GME Accreditation System* being implemented [28], there will be more emphasis on explicit evaluation of discrete milestones and achievement of predefined levels of proficiency. Therefore, developing a valid instrument is paramount. Validity of assessment in itself is contextual and could also be subject to bias of performance, learner, domain, and the interpretation of decisions made based on the assessment data [29]; nonetheless, validity is a reliable measure of sufficient performance and provides an accurate support of evidence to the assessed scenario, and it is widely missing from pre-existing assessment tools [22].

The OSCE assessment has been established as a reliable instrument to assess trainee-residents clinical skills and patient care knowledge. Varkey et al. [30] concluded that it can also be a powerful tool to assess two other competencies, Systems-Based Practice and Practice-Based Learning and Improvement. Short et al. [31] found OSCEs to have a measureable value to assess performance improvement between trainees coming into (incoming/pre-internship) and graduating (outgoing/post-internship) from a program by the end of the year to evaluate program effectiveness on all six competencies. OSCEs have also been found to weakly correlate with the United State Medical Licensing Examination (USMLE) scores in assessing medical graduates skills and knowledge [32]. When Short et al. [31] evaluated OSCEs at the beginning and the end of training; they noted that the improved outcome results might be affected by “retake bias” and familiarity. They also questioned OSCEs’ ability to fully test current actual performance. Further, OSCEs may not necessarily address the shortages the “incoming” testers had in competencies. Using various scenarios to test and evaluate competencies may also introduce bias by confounding with inherent characteristics of each scenario.

In our study, we used one scenario in an attempt to standardize these characteristics although this may cause contamination if trainees took the OSCE and then informed other trainees of upcoming scenarios. Stroud et al. [33] found that using such methodology and scenario did not result in significant differences in performance among their tested residents. The degree of difference between SPs and faculty is expected, as patients may look at things from a different perspective, providing an excellent opportunity for feedback from patients regarding aspects training clinicians can improve upon. Other challenges exist. We realize that generalization of the assessment we conducted in our study to other institutions will be limited by availability of resources, faculty training and information technology infrastructure. Educators and trainees may focus on clinical knowledge and patient care and put lower emphasis on the other competencies. Our study is the first in the U.S. to exclusively assess these two important core competencies through a medical error scenario and to evaluate the use of EMR/EMIR in depth. The use of medical error scenario is a standard practice with noted reliable results in educating and training postgraduate trainees, and our results affirm such findings while minimizing subjective evaluations [33]. The availability of enhancing resources, such as videotaping and simultaneous screenshots, provided an opportunity to assess inter-rater agreement and reliability of the instrument, revisit performance, confirm results, and possibly examine other variables of interest. Furthermore, this work can be extended in future interventional studies that aim at improving proficiency in professionalism, communication, using EMRs/EMIRs and managing medical errors, as well as educating both, faculty and trainees, about standard practices, including detection of medical errors, and providing opportunities for early detection of under-performers, creating opportunities and encouraging feedback for improvements, and recognizing the well-achievers.

## Conclusion

An OSCE-based instrument using a medical error scenario can be used to assess proficiency in professionalism, communication, using EMRs and managing medical errors, and provides significant feedback for the purpose of identifying areas to worm on, improve and help in directing future goals.

## Additional file

Additional file 1: Instrument for Communication skills and Professionalism Assessment (InCoPrA). A copy of the two parts of the assessment instrument that include the Faculty Evaluation of Learner component and the Standardized Patient (SP) Evaluation of Learner forms. (DOC 44 kb)
