# Supplementary material for: Proficiency in identifying, managing and communicating medical errors: feasibility and validity study assessing two core competencies
Source: BMC Med Educ. 2016 Sep 2;16(1):233. doi: 10.1186/s12909-016-0755-5 (PMC5010770; doi:10.1186/s12909-016-0755-5)
Supplement: Additional file 1: — Instrument for Communication skills and Professionalism Assessment (InCoPrA). A copy of the two parts of the assessment instrument that include the Faculty Evaluation of Learner component and the Standardized Patient (SP) Evaluation of Learner forms. (DOC 44 kb) [file 12909_2016_755_MOESM1_ESM.doc]

Faculty Evaluation of Learner

Faculty: Trainee name: Date:

OSCE – Detection and Disclosure of a Medical Error

(Master Checklist)

Please rate the learner in each of the following areas and provide comments at the end about his/her particular strengths and areas for improvement.

1. **SET AN APPROPRIATE CONTEXT FOR DISCUSSION**

**1. Check the box if the learner:**

Established rapport (e.g., introductions, eye contact, body positioning) P.V.: 0

Explained the purpose of the meeting P.V.: 0

**2. How would you rate the learner on his/her ability to set an appropriate context for the discussions?**

Outstanding P.V.: 0

Satisfactory P.V.: 0

Unsatisfactory P.V.: 0

1. **DETECT AND COMMUNICATE A MEDICAL ERROR**

**3. Check the box if the learner:**

Reviewed the medications with the patient P.V.: 0

Explained to the patient what has occurred clearly, fully, without jargon P.V.: 0

Checked for the patient’s understanding of information P.V.: 0

Answered the patient’s questions clearly, directly P.V.: 0

**4. How would you rate the learner on the ability to detect that a medical error has occurred?**

Outstanding P.V.: 0

Satisfactory P.V.: 0

Unsatisfactory P.V.: 0

1. **MANAGEMENT**

**5. Check the box if the learner:**

Queried medical information sources electronically P.V.: 0

Initiated appropriate medication changes P.V.: 0

Initiated appropriate work restrictions P.V.: 0

Arranged for sub-specialty consultation P.V.: 0

**6. Overall, how would you rate this learner’s ability to manage a medical error of this type?**

Outstanding P.V.: 0

Satisfactory P.V.: 0

Unsatisfactory P.V.: 0

1. **EMPATHY**

**7. Check the box if the learner:**

Allowed the patient to express emotions P.V.: 0

Provided words of comfort, arranged for other support as needed P.V.: 0

**8. How would you rate the learner on empathy?**

Outstanding P.V.: 0

Satisfactory P.V.: 0

Unsatisfactory P.V.: 0

1. **USE OF EMR AND EMIR**

**9. Check the box if the learner:**

Queried the electronic health record for medications prescribed P.V.: 0

Sufficiently accesses and reviewed all required records and e-resources P.V.: 0

**10. Overall, how would you rate this learner’s ability to use the electronic medical records and resources in addressing the error?**

Outstanding P.V.: 0

Satisfactory P.V.: 0

Unsatisfactory P.V.: 0

1. **GLOBAL RATING**

**11. Check the box if the learner:**

Patient displayed satisfaction with the professional management (Q10) P.V.: 0

Patient was assured follow-up (how to contact him/her, next communication) P.V.: 0

**12. Overall, how would you rate this learner’s ability to disclose medical errors in a professional manner?**

Outstanding P.V.: 0

Satisfactory P.V.: 0

Unsatisfactory P.V.: 0

**COMMENTS**

**13. Please comment on the learner’s strengths. This information will be shared with the learner.**

________________________________________________________________________________________________________________________________________________________________________________________________________________________________________________________________________________________________________________________________________________________________________

**14. Please comment on the learner’s areas for improvement. This information will be shared with the learner.**

________________________________________________________________________________________________________________________________________________________________________________________________________________________________________________________________________________________________________________________________________________________________________

Patient Evaluation of Learner

SP: Trainee name: Date:

OSCE – Detection and Disclosure of a Medical Error

(Master checklist)

Please rate the learner in each of the following areas and provide comments at the end about his/her particular strengths and areas for improvement.

1. **CONTEXT FOR DISCUSSION**

**1. Check the box if the learner:**

Told me what error had occurred and how P.V.: 0

Told me how it might impact my health and talked about the consequences P.V.: 0

**2. How would you rate the learner on his/her ability to explain the facts regarding the error?**

Outstanding P.V.: 0

Satisfactory P.V.: 0

Unsatisfactory P.V.: 0

1. **HONESTY AND TRUTHFULNESS**

**3. Check the box if the learner:**

Explained the problem freely and directly, without me having to ask

a lot of probing questions to get details P.V.: 0

Did not avoid my questions (was not evasive) P.V.: 0

**4. How would you rate the learner on honesty and truthfulness?**

Outstanding P.V.: 0

Satisfactory P.V.: 0

Unsatisfactory P.V.: 0

1. **EMPATHY**

5**. Check the box if the learner:**

Allowed me to express emotions P.V.: 0

Was sorry and comforting, and responded well to my anger and concerns P.V.: 0

**6. How would you rate the learner on empathy?**

Outstanding P.V.: 0

Satisfactory P.V.: 0

Unsatisfactory P.V.: 0

1. **CLOSURE**

**7. Check the box if the learner:**

Assured follow-up (how to contact him/her, when to expect next

communication) P.V.: 0

Took appropriate leave (handshake, avoided “hasty retreat”) P.V.: 0

**8. How would you rate the learner on providing closure to the discussion?**

Outstanding P.V.: 0

Satisfactory P.V.: 0

Unsatisfactory P.V.: 0

1. **TRUSHWORTHINESS/GLOBAL RATING**

**9. Check the box if the learner:**

Was able to manage my problems in a professional/compassionate manner P.V.: 0

Spent sufficient time with me to address my concerns (disclose the error) P.V.: 0

**10. Given this discussion, how comfortable are you with entrusting a loved one’s care to this learner?**

Comfortable P.V.: 0

Somewhat comfortable P.V.: 0

Not at all comfortable P.V.: 0

**COMMENTS**

**11. Please comment on the learner’s strengths. This information will be shared with the learner.**

______________________________________________________________________________________________________________________________________________________________________________________________________________________________________________________________________________

**12. Please comment on the learner’s areas for improvement. This information will be shared with the learner.**

_____________________________________________________________________________________________________________________________________________________________________________________________________________________________________________________________________
